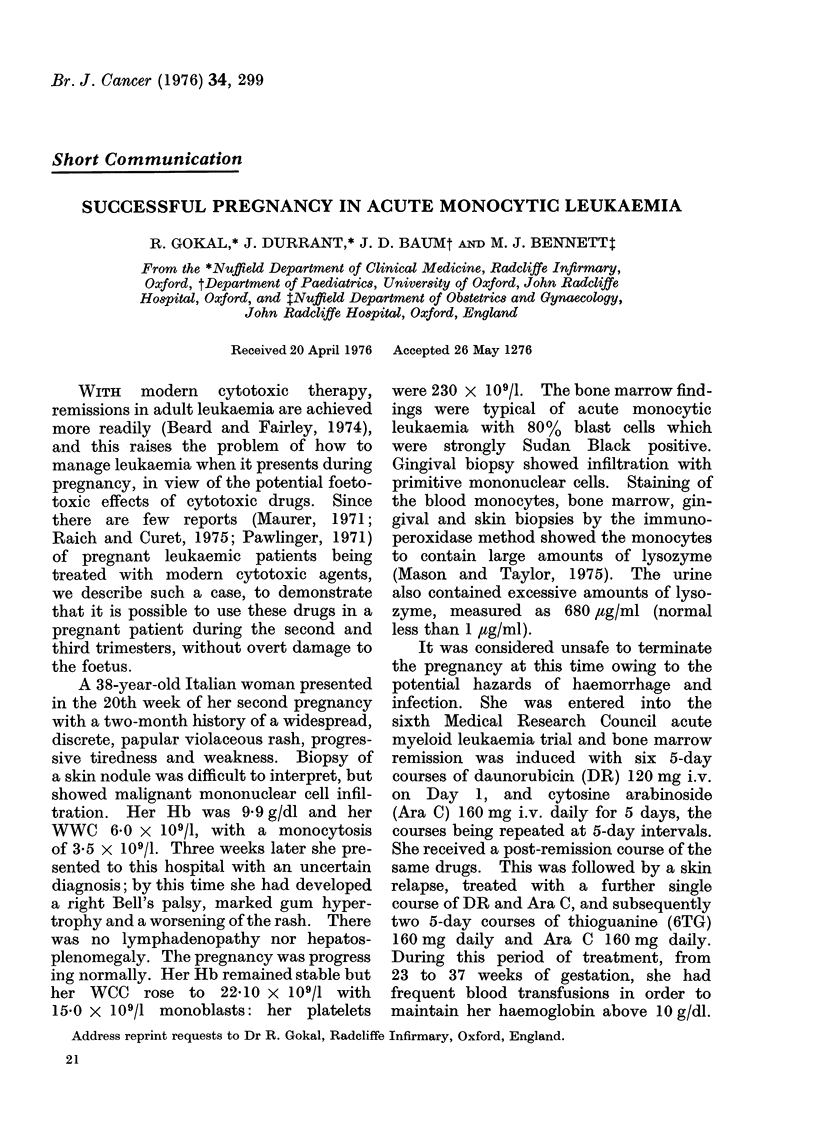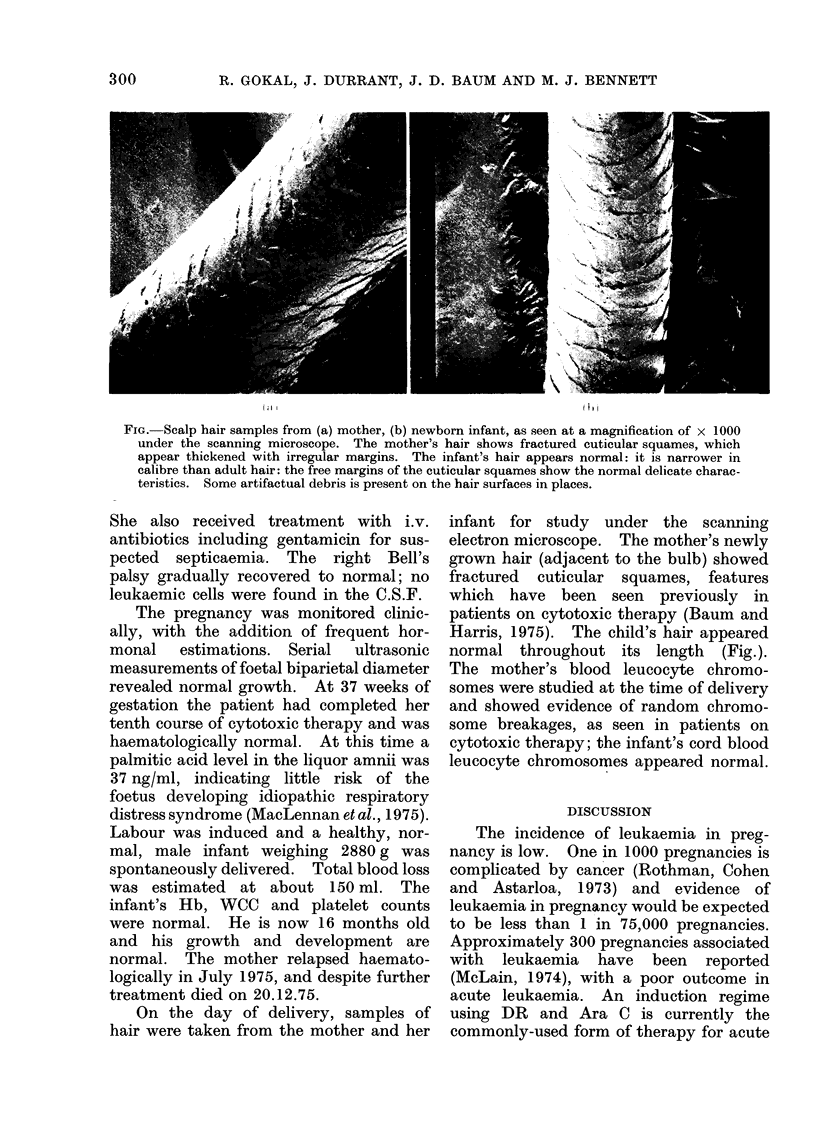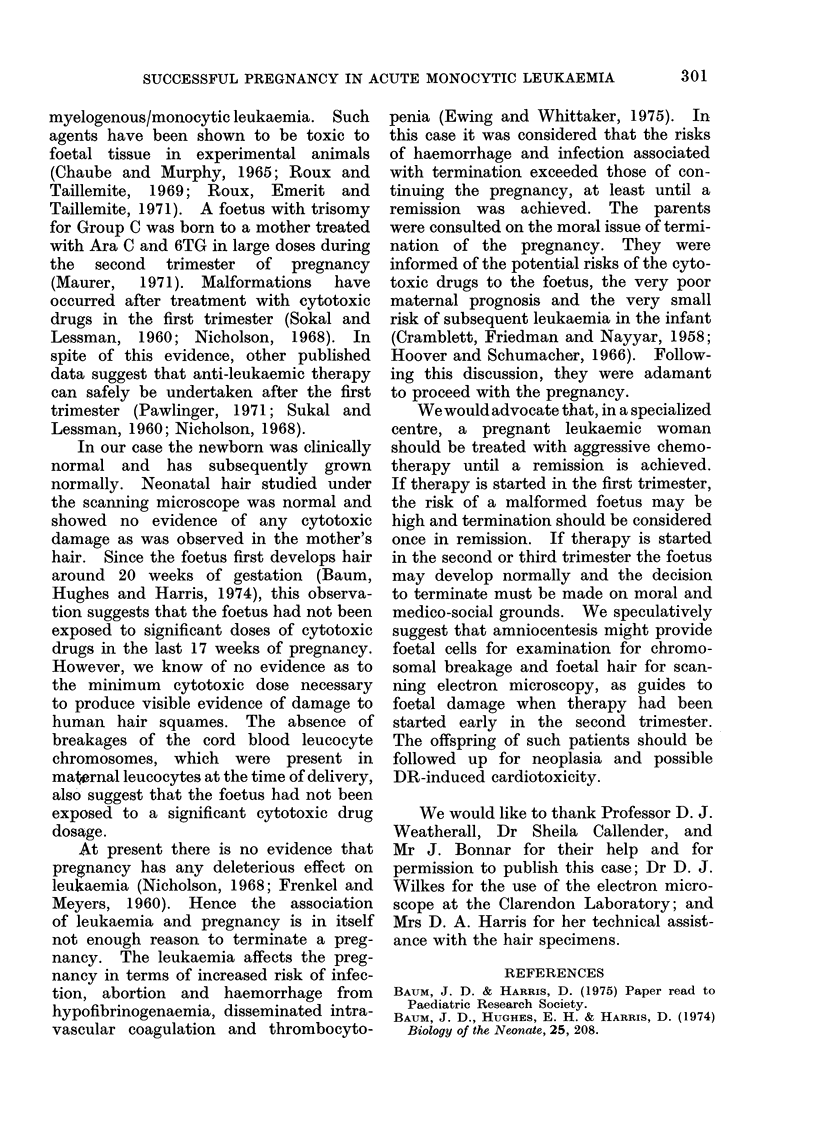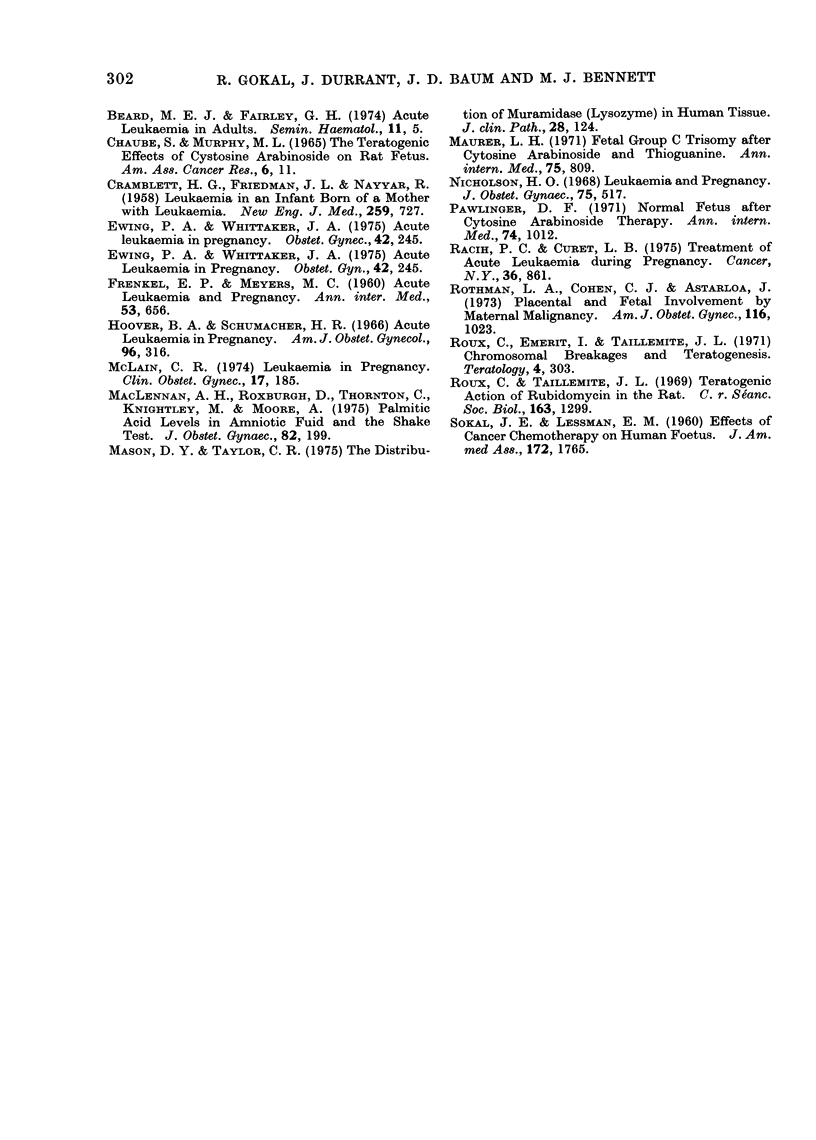# Successful pregnancy in acute monocytic leukaemia.

**DOI:** 10.1038/bjc.1976.167

**Published:** 1976-09

**Authors:** R. Gokal, J. Durrant, J. D. Baum, M. J. Bennett

## Abstract

**Images:**


*Br. J. Cancer* (1976) **34**, 299

### *Short Communication*

### SUCCESSFUL PREGNANCY IN ACUTE MONOCYTIC LEUKAEMIA

R. GOKAL,* J. DURRANT,* J. D. BAUM† AND M. J. BENNETT‡

*From the *Nuffield Department of Clinical Medicine, Radcliffe Infirmary, Oxford, †Department of Paediatrics, University of Oxford, John Radcliffe Hospital, Oxford, and ‡Nuffield Department of Obstetrics and Gynaecology, John Radcliffe Hospital, Oxford, England*

Received 20 April 1976 Accepted 26 May 1276

WITH modern cytotoxic therapy, remissions in adult leukaemia are achieved more readily (Beard and Fairley, 1974), and this raises the problem of how to manage leukaemia when it presents during pregnancy, in view of the potential foeto-toxic effects of cytotoxic drugs. Since there are few reports (Maurer, 1971; Raich and Curet, 1975; Pawlinger, 1971) of pregnant leukaemic patients being treated with modern cytotoxic agents, we describe such a case, to demonstrate that it is possible to use these drugs in a pregnant patient during the second and third trimesters, without overt damage to the foetus.

A 38-year-old Italian woman presented in the 20th week of her second pregnancy with a two-month history of a widespread, discrete, papular violaceous rash, progressive tiredness and weakness. Biopsy of a skin nodule was difficult to interpret, but showed malignant mononuclear cell infiltration. Her Hb was 9·9 g/dl and her WWC $6 \cdot 0 \times 10^9$/l, with a monocytosis of $3 \cdot 5 \times 10^9$/l. Three weeks later she presented to this hospital with an uncertain diagnosis; by this time she had developed a right Bell's palsy, marked gum hypertrophy and a worsening of the rash. There was no lymphadenopathy nor hepatosplenomegaly. The pregnancy was progress ing normally. Her Hb remained stable but her WCC rose to $22 \cdot 10 \times 10^9$/l with $15 \cdot 0 \times 10^9$/l monoblasts: her platelets were $230 \times 10^9$/l. The bone marrow findings were typical of acute monocytic leukaemia with 80% blast cells which were strongly Sudan Black positive. Gingival biopsy showed infiltration with primitive mononuclear cells. Staining of the blood monocytes, bone marrow, gingival and skin biopsies by the immunoperoxidase method showed the monocytes to contain large amounts of lysozyme (Mason and Taylor, 1975). The urine also contained excessive amounts of lysozyme, measured as 680 μg/ml (normal less than 1 μg/ml).

It was considered unsafe to terminate the pregnancy at this time owing to the potential hazards of haemorrhage and infection. She was entered into the sixth Medical Research Council acute myeloid leukaemia trial and bone marrow remission was induced with six 5-day courses of daunorubicin (DR) 120 mg i.v. on Day 1, and cytosine arabinoside (Ara C) 160 mg i.v. daily for 5 days, the courses being repeated at 5-day intervals. She received a post-remission course of the same drugs. This was followed by a skin relapse, treated with a further single course of DR and Ara C, and subsequently two 5-day courses of thioguanine (6TG) 160 mg daily and Ara C 160 mg daily. During this period of treatment, from 23 to 37 weeks of gestation, she had frequent blood transfusions in order to maintain her haemoglobin above 10 g/dl.

Address reprint requests to Dr R. Gokal, Radcliffe Infirmary, Oxford, England.

(a) (b)

FIG.—Scalp hair samples from (a) mother, (b) newborn infant, as seen at a magnification of × 1000 under the scanning microscope. The mother's hair shows fractured cuticular squames, which appear thickened with irregular margins. The infant's hair appears normal: it is narrower in calibre than adult hair: the free margins of the cuticular squames show the normal delicate characteristics. Some artifactual debris is present on the hair surfaces in places.

She also received treatment with i.v. antibiotics including gentamicin for suspected septicaemia. The right Bell's palsy gradually recovered to normal; no leukaemic cells were found in the C.S.F.

The pregnancy was monitored clinically, with the addition of frequent hormonal estimations. Serial ultrasonic measurements of foetal biparietal diameter revealed normal growth. At 37 weeks of gestation the patient had completed her tenth course of cytotoxic therapy and was haematologically normal. At this time a palmitic acid level in the liquor amnii was 37 ng/ml, indicating little risk of the foetus developing idiopathic respiratory distress syndrome (MacLennan *et al.*, 1975). Labour was induced and a healthy, normal, male infant weighing 2880 g was spontaneously delivered. Total blood loss was estimated at about 150 ml. The infant's Hb, WCC and platelet counts were normal. He is now 16 months old and his growth and development are normal. The mother relapsed haematologically in July 1975, and despite further treatment died on 20.12.75.

On the day of delivery, samples of hair were taken from the mother and her infant for study under the scanning electron microscope. The mother's newly grown hair (adjacent to the bulb) showed fractured cuticular squames, features which have been seen previously in patients on cytotoxic therapy (Baum and Harris, 1975). The child's hair appeared normal throughout its length (Fig.). The mother's blood leucocyte chromosomes were studied at the time of delivery and showed evidence of random chromosome breakages, as seen in patients on cytotoxic therapy; the infant's cord blood leucocyte chromosomes appeared normal.

### DISCUSSION

The incidence of leukaemia in pregnancy is low. One in 1000 pregnancies is complicated by cancer (Rothman, Cohen and Astarloa, 1973) and evidence of leukaemia in pregnancy would be expected to be less than 1 in 75,000 pregnancies. Approximately 300 pregnancies associated with leukaemia have been reported (McLain, 1974), with a poor outcome in acute leukaemia. An induction regime using DR and Ara C is currently the commonly-used form of therapy for acute myelogenous/monocytic leukaemia. Such agents have been shown to be toxic to foetal tissue in experimental animals (Chaube and Murphy, 1965; Roux and Taillemite, 1969; Roux, Emerit and Taillemite, 1971). A foetus with trisomy for Group C was born to a mother treated with Ara C and 6TG in large doses during the second trimester of pregnancy (Maurer, 1971). Malformations have occurred after treatment with cytotoxic drugs in the first trimester (Sokal and Lessman, 1960; Nicholson, 1968). In spite of this evidence, other published data suggest that anti-leukaemic therapy can safely be undertaken after the first trimester (Pawlinger, 1971; Sukal and Lessman, 1960; Nicholson, 1968).

In our case the newborn was clinically normal and has subsequently grown normally. Neonatal hair studied under the scanning microscope was normal and showed no evidence of any cytotoxic damage as was observed in the mother's hair. Since the foetus first develops hair around 20 weeks of gestation (Baum, Hughes and Harris, 1974), this observation suggests that the foetus had not been exposed to significant doses of cytotoxic drugs in the last 17 weeks of pregnancy. However, we know of no evidence as to the minimum cytotoxic dose necessary to produce visible evidence of damage to human hair squames. The absence of breakages of the cord blood leucocyte chromosomes, which were present in maternal leucocytes at the time of delivery, also suggest that the foetus had not been exposed to a significant cytotoxic drug dosage.

At present there is no evidence that pregnancy has any deleterious effect on leukaemia (Nicholson, 1968; Frenkel and Meyers, 1960). Hence the association of leukaemia and pregnancy is in itself not enough reason to terminate a pregnancy. The leukaemia affects the pregnancy in terms of increased risk of infection, abortion and haemorrhage from hypofibrinogenaemia, disseminated intravascular coagulation and thrombocytopenia (Ewing and Whittaker, 1975). In this case it was considered that the risks of haemorrhage and infection associated with termination exceeded those of continuing the pregnancy, at least until a remission was achieved. The parents were consulted on the moral issue of termination of the pregnancy. They were informed of the potential risks of the cytotoxic drugs to the foetus, the very poor maternal prognosis and the very small risk of subsequent leukaemia in the infant (Cramblett, Friedman and Nayyar, 1958; Hoover and Schumacher, 1966). Following this discussion, they were adamant to proceed with the pregnancy.

We would advocate that, in a specialized centre, a pregnant leukaemic woman should be treated with aggressive chemotherapy until a remission is achieved. If therapy is started in the first trimester, the risk of a malformed foetus may be high and termination should be considered once in remission. If therapy is started in the second or third trimester the foetus may develop normally and the decision to terminate must be made on moral and medico-social grounds. We speculatively suggest that amniocentesis might provide foetal cells for examination for chromosomal breakage and foetal hair for scanning electron microscopy, as guides to foetal damage when therapy had been started early in the second trimester. The offspring of such patients should be followed up for neoplasia and possible DR-induced cardiotoxicity.

We would like to thank Professor D. J. Weatherall, Dr Sheila Callender, and Mr J. Bonnar for their help and for permission to publish this case; Dr D. J. Wilkes for the use of the electron microscope at the Clarendon Laboratory; and Mrs D. A. Harris for her technical assistance with the hair specimens.

REFERENCES

Baum, J. D. & Harris, D. (1975) Paper read to Paediatric Research Society.

Baum, J. D., Hughes, E. H. & Harris, D. (1974) *Biology of the Neonate*, **25**, 208.

BEARD, M. E. J. & FAIRLEY, G. H. (1974) Acute Leukaemia in Adults. *Semin. Haematol.*, **11**, 5.

CHAUBE, S. & MURPHY, M. L. (1965) The Teratogenic Effects of Cystosine Arabinoside on Rat Fetus. *Am. Ass. Cancer Res.*, **6**, 11.

CRAMBLETT, H. G., FRIEDMAN, J. L. & NAYYAR, R. (1958) Leukaemia in an Infant Born of a Mother with Leukaemia. *New Eng. J. Med.*, **259**, 727.

EWING, P. A. & WHITTAKER, J. A. (1975) Acute leukaemia in pregnancy. *Obstet. Gynec.*, **42**, 245.

EWING, P. A. & WHITTAKER, J. A. (1975) Acute Leukaemia in Pregnancy. *Obstet. Gyn.*, **42**, 245.

FRENKEL, E. P. & MEYERS, M. C. (1960) Acute Leukaemia and Pregnancy. *Ann. inter. Med.*, **53**, 656.

HOOVER, B. A. & SCHUMACHER, H. R. (1966) Acute Leukaemia in Pregnancy. *Am. J. Obstet. Gynecol.*, **96**, 316.

McLAIN, C. R. (1974) Leukaemia in Pregnancy. *Clin. Obstet. Gynec.*, **17**, 185.

MACLENNAN, A. H., ROXBURGH, D., THORNTON, C., KNIGHTLEY, M. & MOORE, A. (1975) Palmitic Acid Levels in Amniotic Fuid and the Shake Test. *J. Obstet. Gynaec.*, **82**, 199.

MASON, D. Y. & TAYLOR, C. R. (1975) The Distribution of Muramidase (Lysozyme) in Human Tissue. *J. clin. Path.*, **28**, 124.

MAURER, L. H. (1971) Fetal Group C Trisomy after Cytosine Arabinoside and Thioguanine. *Ann. intern. Med.*, **75**, 809.

NICHOLSON, H. O. (1968) Leukaemia and Pregnancy. *J. Obstet. Gynaec.*, **75**, 517.

PAWLINGER, D. F. (1971) Normal Fetus after Cytosine Arabinoside Therapy. *Ann. intern. Med.*, **74**, 1012.

RACIH, P. C. & CURET, L. B. (1975) Treatment of Acute Leukaemia during Pregnancy. *Cancer, N.Y.*, **36**, 861.

ROTHMAN, L. A., COHEN, C. J. & ASTARLOA, J. (1973) Placental and Fetal Involvement by Maternal Malignancy. *Am. J. Obstet. Gynec.*, **116**, 1023.

ROUX, C., EMERIT, I. & TAILLEMITE, J. L. (1971) Chromosomal Breakages and Teratogenesis. *Teratology*, **4**, 303.

ROUX, C. & TAILLEMITE, J. L. (1969) Teratogenic Action of Rubidomycin in the Rat. *C. r. Séanc. Soc. Biol.*, **163**, 1299.

SOKAL, J. E. & LESSMAN, E. M. (1960) Effects of Cancer Chemotherapy on Human Foetus. *J. Am. med Ass.*, **172**, 1765.